# Supplementary material for: A Cross between Bread Wheat and a 2D(2R) Disomic Substitution Triticale Line Leads to the Formation of a Novel Disomic Addition Line and Provides Information of the Role of Rye Secalins on Breadmaking Characteristics
Source: Int J Mol Sci. 2020 Nov 10;21(22):8450. doi: 10.3390/ijms21228450 (PMC7696169; doi:10.3390/ijms21228450)
Supplement: Supplementary file 1 [file ijms-21-08450-s001.pdf]

Table S1. Qualitative parameters determined in two different growing seasons (2016-2017 and 2017-2018).

Genotype	N11	N11^{XY7}	XY7	N11	N11^{XY7}	XY7
**Season**		2016-17			2017-18	
**Test weight (kg/hL)**	81.0a	79.5a	69.0b	84.1a	80.6a	69.3b
**Hardness**	101	106	112	76	85	88
**Flour protein content (% d.m.)**	10.4a	14.6b	11.4a	10.8a	13.7b	10.6a
**Gluten Index**	91	100	69	82	99	59
**Alveograph test**						
**P (mm)**	108a	82ab	58b	92a	83ab	62b
**L (mm)**	63a	169b	54a	96a	181c	56b
**P/L**	1.71	0.49	1.07	0.96	0.46	1.11
**W (Joules x 10^{-4})**	223a	474c	97b	239a	458c	106b
**Farinograph test**						
**Water abs. (%)**	61.1a	60.8a	53.3b	60.6a	60.7a	54.8b
**Dough development time (min)**	1.6a	10.3b	1.3a	4.0a	9.1b	1.4a
**Dough stability (min)**	3.9a	11.7b	3.4a	6.7a	11.9b	3.7a
**Degree of softening (BU)**	73a	58a	119b	51a	43a	119b
**FQN (mm)**	58a	172c	46b	87a	170c	53b

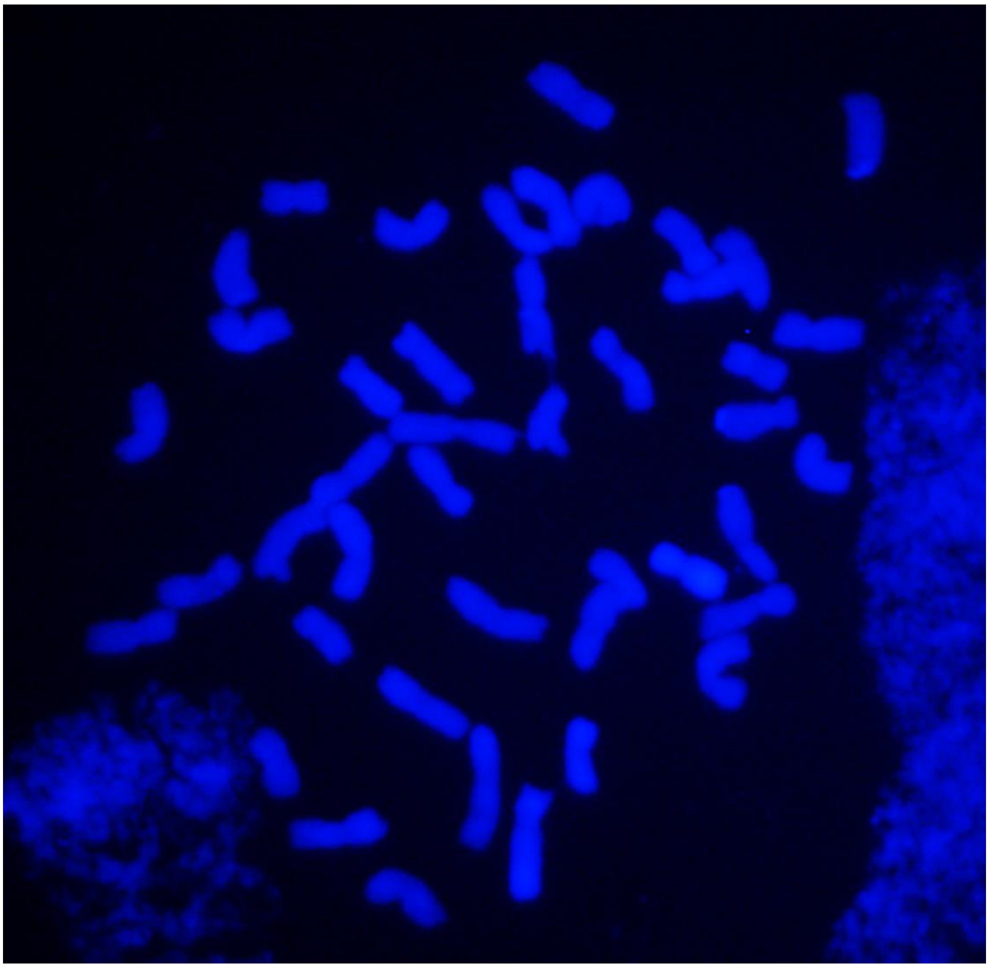

Figure S1. Metaphase spread of N11^{XY7} line counterstained with DAPI for chromosomes counting. N11^{XY7} chromosomes number 2n=44.
